# Nonsteroidal Anti-Inflammatory Drugs Prevent Vincristine-Dependent Cancer-Associated Fibroblasts Formation

**DOI:** 10.3390/ijms20081941

**Published:** 2019-04-20

**Authors:** Marta Ewelina Wawro, Katarzyna Sobierajska, Wojciech Michał Ciszewski, Jolanta Niewiarowska

**Affiliations:** Department of Molecular Cell Mechanisms, Medical University of Lodz, Mazowiecka 6/8, 92-215 Lodz, Poland; marta.wawro@umed.lodz.pl (M.E.W.); wojciech.ciszewski@umed.lodz.pl (W.M.C.)

**Keywords:** EndMT, immunomodulation, CAFs, colon cancer, microtubules, IL-6, TGF-βs, vincristine, aspirin, ibuprofen

## Abstract

Vincristine is used in the clinical treatment of colon cancer, especially in patients diagnosed in the advanced phase of cancer development. Unfortunately, similar to other agents used during antitumor therapy, vincristine might induce chemoresistance. Studies of this process focus mainly on the analysis of the molecular mechanisms within cancer, usually ignoring the role of stromal cells. Our present findings confirm that vincristine stimulates the secretion of tumor growth factors class beta and interleukin-6 from cancer-associated fibroblasts as a result of paracrine stimulation by cancer cells. Based on alterations in morphology, modulation of capillary formation, and changes in endothelial and mesenchymal marker profile, our findings demonstrate that higher levels of tumor growth factor-βs and interleukin-6 enhance cancer-associated fibroblast-like cell formation through endothelial–mesenchymal transition and that nonsteroidal anti-inflammatory drug treatment (aspirin and ibuprofen) is able to inhibit this phenomenon. The process appears to be regulated by the rate of microtubule polymerization, depending on β-tubulin composition. While higher levels of tubulin-β2 and tubulin-β4 caused slowed polymerization and reduced the level of factors secreted to the extracellular matrix, tubulin-β3 induced the opposite effect. We conclude that nonsteroidal anti-inflammatory drugs should be considered for use during vincristine monotherapy in the treatment of patients diagnosed with colorectal cancer.

## 1. Introduction

Besides malignant cells, solid tumors also consist of numerous stromal cells, such as fibroblasts, endothelial cells, and cells of the immune system located in the tumor niche [1]. Tumor progression is promoted by both the proliferation of cancer cells and their interaction with stromal cells [1,2]. Cancer cells modulate the stroma of the tumor in a paracrine manner. The released cytokines and growth factors such as proteins from Transforming Growth Factor-beta family (TGF-βs) and interleukins remodel the components of the tumor niche to support cancer development. In return, cells located in the tumor niche regulate cancer progression through secreted immunomodulators, thus stimulating the migration and invasion capability of tumor cells [2,3]. Furthermore, factors released by stromal cells might induce an alteration in other cells located in the cancer niche [2].

Cancer-associated fibroblasts (CAFs) are a heterogenic group of stromal cells which transdifferentiate from numerous other cell types, including the endothelium. One of the main sources of CAFs is the endothelium of microvessels located in the tumor niche, which undergoes an endothelial–mesenchymal transition (EndMT) [4]. CAFs are large, strongly elongated spindle-shaped mesenchymal cells described as the main inductor of cancer invasion ability and metastasis through secreted immunomodulators such as TGF-β [5]. In addition, CAFs might indirectly regulate cancer progression by altering the function of other stromal cells [5]. Tumor cells are also able to regulate the profile of the factors secreted by CAFs that stimulate cancer development; for example, interleukin-6 (IL-6) released from CAFs may modulate tumor angiogenesis [6].

A commonly used therapy for patients diagnosed with an advanced stage of colon cancer is vincristine (VIN), which destabilizes the structures of tubulins in microtubules by binding to them [7]. By this inhibition of the microtubule structure assembly, vincristine arrests mitosis in the metaphase. However, similar to other antitumor cytostatics, vincristine might also induce chemoresistance. Previous studies have focused mainly on the role of cancer cells and molecular processes regulating the induction of chemoresistance within these cells and have neglected the role of stromal cells.

Currently, nonsteroidal anti-inflammatory drugs (NSAIDs) commonly recommended for patients with coronary heart disease are given in the management of patients with recognized cancer [8]. Analysis of epidemiological data demonstrated inversely related correlation between breast, colorectal, and lung cancers incidents and anti-inflammatory drugs including aspirin (AsA) and ibuprofen (IBU) [9,10,11]. Moreover, long-term NSAID-treated patients had dramatically lower mortality rates than non-NSAID-treated [12]. Nonsteroidal anti-inflammatory drugs were initially used to counteract activation of the blood coagulation system and reduce the risk of venous thromboembolism observed in the course of malignancy development. Recently, nonsteroidal anti-inflammatory drugs have also been recommended for the prevention of colorectal cancer development [13]. However, their molecular mechanisms regulated by nonsteroidal anti-inflammatory drugs in preventing the cancer development and the organization of its niche are virtually unknown.

The present study focuses on the effect of interaction between colon cancer and cancer-associated fibroblast-like (CAF-like) cells in the vincristine-induced modulation of endothelial cells isolated from microvessels. It clarifies the role of paracrine interaction between colon cancer and cancer-associated fibroblast-like cells in the transdifferentiation of endothelial cells induced by vincristine. It also investigates the role of NSAIDs in the prevention of EndMT-dependent cancer-associated fibroblast-like cell formation via the modulation of microtubule polymerization.

## 2. Results

### 2.1. The Interaction between Colon Cancer Cells and CAF-like Cells Treated with Vincristine Induce Mesenchymal Transdifferentiation of Endothelium and Fibroblasts

**Figure 1:**
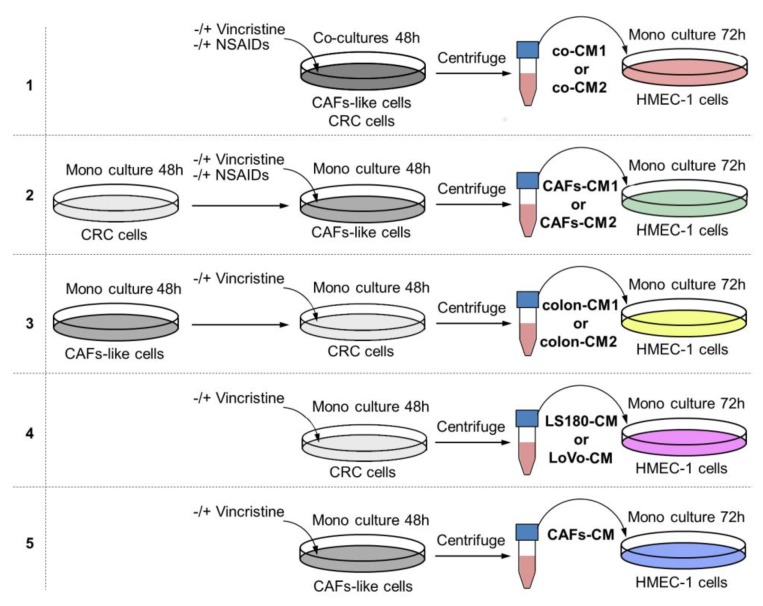
The research scheme for determining the role of NSAIDs in preventing vincristine-dependent CAF formation. In each experiment, the conditioned medium (CM) obtained from preinvasive colon cancer cells (LS180) was called CM1, and from invasive cell line LoVo, CM2. Each CM was labeled with other colors according to the scheme. Model **1**. The coculture of colon cancer cell lines and CAF-like cells were maintained during 48 h and, if necessary, treated with vincristine (VIN) during the final 24 h. The CM was collected, centrifuged, and added to HMEC-1 cells grown in MCDB-131 medium in a 1:3 ratio for 72 h. In some experiments, NSAIDs (AsA or IBU) were added to the coculture cells for the final 24 h. Model **2**. The colon cancer cell lines were maintained in DMEM medium for 48 h. Next, CM was collected, centrifuged, and added to CAF-like cells. CAFs were treated, if necessary, with vincristine (VIN) during the final 24 h. Then, CM was collected, centrifuged, and finally added to HMEC-1 cells grown in MCDB-131 medium in a 1:3 ratio for 72 h. In some experiments, NSAIDs (AsA or IBU) were added to the CAF-like cells for the last 24 h. Model **3**. The CAF-like cells were grown for 48 h. Next, CM was collected, centrifuged, and added to colon cancer cell lines. Colon cancer cells were treated, if necessary, with vincristine (VIN) during the final 24 h. Then, CM was collected, centrifuged, and added to HMEC-1 cells grown in MCDB-131 medium in the 1:3 ratio for 72 h. Model **4**. The colon cancer cell lines were maintained in DMEM medium for 48 h. The CM was collected, centrifuged, and finally added to HMEC-1 cells grown in MCDB-131 medium in the 1:3 ratio for 72 h. In some experiments, vincristine was added to the colon cancer cells for the last 24 h. Model **5**. The CAF-like cells were maintained in DMEM medium for 48 h. Then, CM was collected, centrifuged, and finally added to HMEC-1 cells grown in MCDB-131 medium in the 1:3 ratio for 72 h. In some experiments, vincristine was added to the CAF-like cells for the final 24 h.

To determine whether the interaction between colon cancer cells and CAFs might alter the effects of vincristine treatment by modulating the cancer niche, a coculture of colon cancer and CAF-like cells (Figure 1, model 1) was prepared and treated, if necessary, with vincristine. To analyze the effect of tumor development stage in the studied process, two colon cancer cell lines were used with conditioned media obtained from preinvasive (LS180-CM1) and invasive (LoVo-CM2) colon cancer stages.

**Figure 2:**
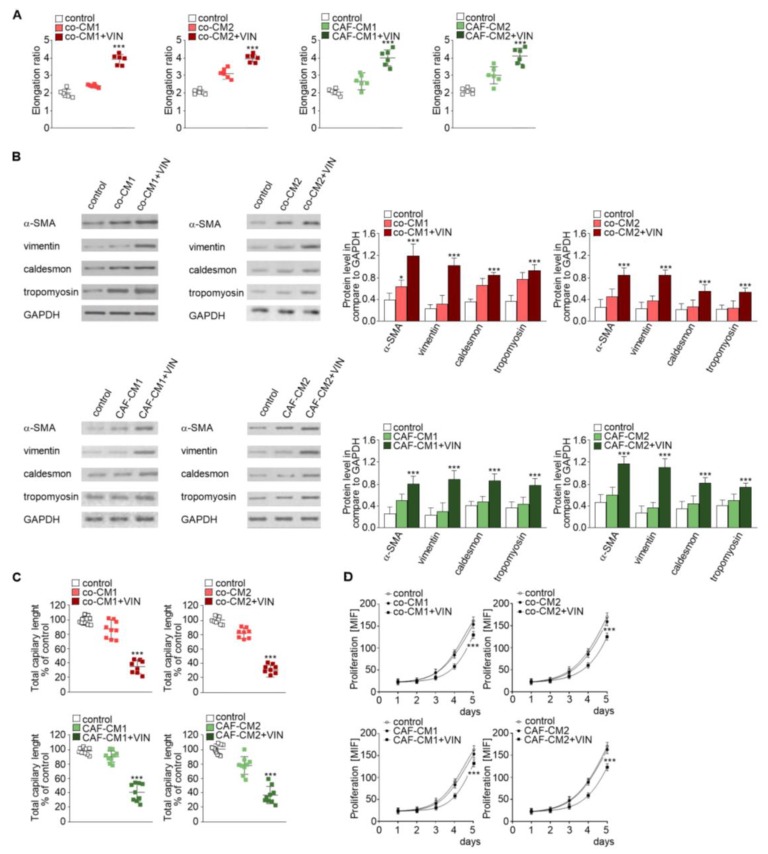
Mesenchymal transdifferentiation in HMEC-1 is modulated by vincristine-treated CAF-like cells. HMEC-1 cells were cultured in medium supplemented with CM isolated from coculture of CAF-like cells and colon cancer (LS180—co-CM1 or LoVo—co-CM2) or CAF-like cells maintained in CM colon cancer cells (LS180—CAFs-CM1 or LoVo—CAFs-CM2) and treated, if necessary, with vincristine (+VIN). Then, elongation ratio (*n* = 6) (**A**), level of contraction proteins (caldesmon, tropomyosin), vimentin, and α-SMA (Western blot) (**B**), capillary assay (*n* = 9) (**C**), and proliferation ability (**D**) were analyzed. In Western blot assay, GAPDH was used as the loading control. The results are provided as means ± SD (*n* = 3); * *p* < 0.05, *** *p* < 0.005. The blots are representative of three independent experiments.

Firstly, we analyzed the effect of CM obtained from coculture (co-CM1 or co-CM2) on HMEC-1 cell behavior. The HMEC-1 maintained in the co-CM1 or co-CM2 were found to be slightly elongated (more than 1.3 times) compared to the standard medium (Figure 2A, left panel, Figure S1A, upper panel). This effect was strongly marked (about twofold) when the cells were maintained in CM from coculture treated with vincristine (co-CM1 + VIN or co-CM2 + VIN) compared to controls. Surprisingly, the results were not dependent on colon cancer development stage: preinvasive LS180 (CM1) or invasive LoVo (CM2) (Figure 2A). The CM obtained from the monoculture controls, colon cancer cells (Figure 1 model 4), or CAF-like cells (Figure 1, model 5) treated with vincristine did not demonstrate any modulation of cell shape (data not shown) compared to control cells grown in MCDB medium.

Next, to examine which cell type (colon cancer or CAF-like cells) contributes to the observed change and whether the paracrine interaction of both cell types is necessary for cell elongation, two CM models were performed. Firstly, CAF-like cells treated with vincristine in CM were obtained from colon cancer cells (preinvasive—LS180-CM1, or invasive—LoVo-CM2). Following this, CM obtained from CAF-like cells (CAFs-CM1 + VIN or CAFs-CM2 + VIN) were collected, centrifuged, and added to HMEC-1 culture in a 1:3 ratio (Figure 1, model 2). The HMEC-1 cells grown in CAFs-CM1 + VIN or CAFs-CM2 + VIN (Figure 2A, right panel, Figure S1A, lower panel) showed similar elongation ratio to the cells maintained by co-CM1 + VIN or co-CM2 + VIN. 

Western blot analysis found that the elongation of cells treated with co-CM1 + VIN, co-CM2 + VIN, CAFs-CM1 + VIN, or CAFs-CM2 + VIN was accompanied by CAF-like cell formation via EndMT. Cells maintained in co-CM1 + VIN, co-CM2 + VIN, CAFs-CM1 + VIN, or CAFs-CM2 + VIN showed elevated expression of α-SMA, vimentin, and contraction proteins in comparison to control cells: more than 3.0-fold, 2.6-fold, and 2.0-fold, respectively (Figure 2B). Additionally, vincristine treatment induced lower capillary formation ability by HMEC-1 cells, which was specific to HMEC-1 cells grown in each analyzed CM (Figure 2C, Figure S1B). Finally, we demonstrated that HMEC-1 maintained in co-CM1 + VIN, co-CM2 + VIN, CAFs-CM1 + VIN, or CAFs-CM2 + VIN were characterized by a decrease of proliferation ability (Figure 2D). 

**Figure 3:**
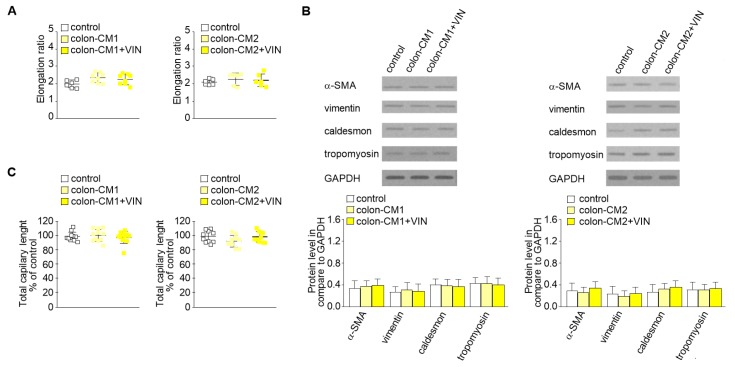
Mesenchymal transdifferentiation in HMEC-1 is not induced by CM from colon cancer cells treated with vincristine. HMEC-1 cells were cultured in medium supplemented with CM isolated from colon cancer cell lines maintained in CM CAFs and treated, if necessary, with vincristine (+VIN). Then, elongation ratio (*n* = 6) (**A**), level of contraction proteins (caldesmon, tropomyosin), vimentin, and α-SMA (Western blot) (**B**), and capillary assay (*n* = 9) (**C**) were analyzed. In the Western blot assay, GAPDH was used as the loading control. The results are provided as means ± SD (*n* = 3). The blots are representative of three independent experiments.

Then, to study whether vincristine-treated colon cancer cells undergoing the paracrine impact of CAFs might regulate EndMT in HMEC-1 cells, a third cellular model was established. Briefly, the colon cancer cells isolated from noninvasive (LS180-CM1) or invasive (LoVo-CM2) stages of cancer development were grown in CM obtained from CAFs (Figure 1, model 3). Then, the conditioned medium was collected, centrifuged, and added to HMEC-1 culture in 1:3 ratio. Our studies showed that medium enriched with colon-CM1 or colon-CM2 did not show any effect on HMEC-1 cell behavior (Figure 3). Neither cell morphology nor elongation ratio was changed with regard to controls (Figure 3A). No differences in vimentin, α-SMA, caldesmon, or tropomyosin levels were found compared to controls (Figure 3B). Additionally, the ability for capillary formation remained unchanged (Figure 3C). 

### 2.2. CAF-like Cell Secretion of IL-6, TGF-β1, and TGF-β2 Increases after Vincristine Treatment

**Figure 4:**
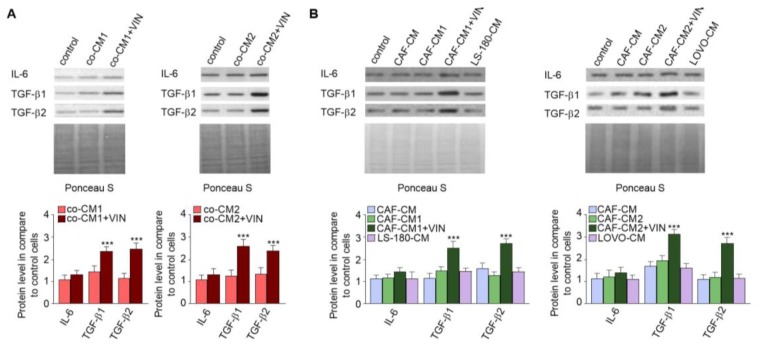
Vincristine enhances secretion of TGF-βs and IL-6 in CAF-like cells through its paracrine interaction with colon cancer cells. Level of TGF-βs (TGF-β1 and TGF-β2) and IL-6 in CM isolated from coculture of CAF-like cells and colon cancer (**A**) or CAF-like cells maintained in colon cancer cell CM (and treated, if necessary, with vincristine) were studied (**B**) by Western blot. The results are provided as means ± SD (*n* = 3); *** *p* < 0.005. As the control of loading, the Ponceau Staining was shown. The blots are representative of three independent experiments.

It has been known that CAFs are differentiated from other cell types thought cytokines and growth factor stimulation [14,15]. Therefore, the secretion of cytokines and growth factors was examined in each studied CM. The immunochemical analysis revealed increased levels of IL-6 (1.2-fold), TGF-β1 (2.5-fold), and TGF-β2 (2.4-fold) in the cocultures treated with vincristine (co-CM1 or co-CM2) (Figure 4A). Similar changes were observed in the profile of the factors secreted by CAF-like cells grown in the CM obtained from colon cancer cells and treated with vincristine (CAFs-CM1 + VIN or CAFs-CM2 + VIN) (Figure 4B); however, the values for LS180-CM, LoVo-CM, colon-CM1, and colon-CM2 were similar to those observed in control cells (Figure 4B). Additionally, the control of loading the blots was labeled by Ponceau Red.

### 2.3. Nonsteroidal Anti-Inflammatory Drugs (NSAIDs) Prevent Vincristine-Dependent EndMT

**Figure 5:**
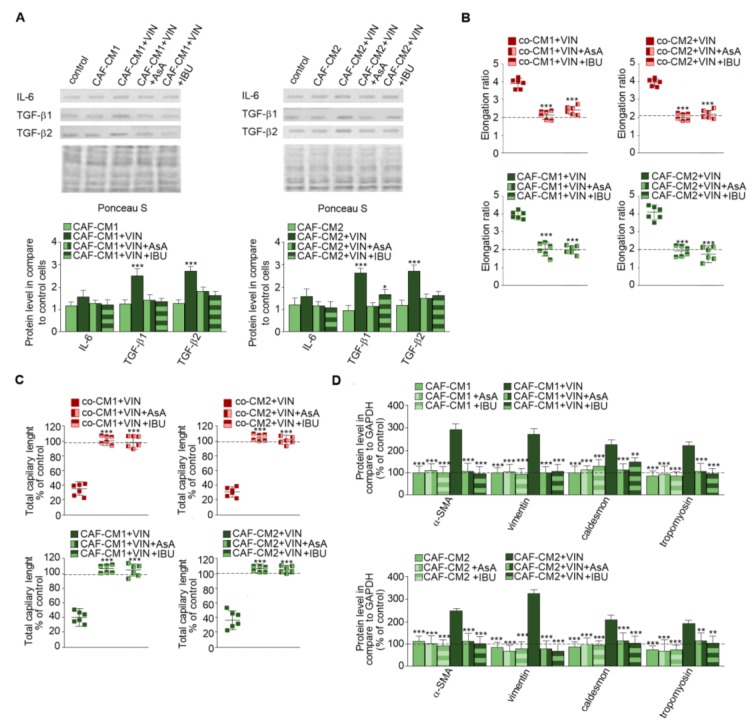
NSAIDs inhibit the effect of vincristine-induced EndMT stimulation. Level of TGF-βs (TGF-β1 and TGF-β2) and IL-6 in CM isolated from coculture of CAF-like cells and colon cancer or CAF-like cells maintained in colon cancer cell CM treated, if necessary, with vincristine (**A**) were studied by Western blot. The results are provided as means ± SD (*n* = 3); * *p* < 0.05, *** *p* < 0.005. The blots are representative of three independent experiments. As the control of loading, the Ponceau Staining was shown. Then, HMEC-1 cells were cultured in medium supplemented with CM isolated from coculture of CAF-like cells and colon cancer or CAF-like cells maintained in colon cancer cell CM and treated, if necessary, with vincristine (+VIN) or NSAIDs (IBU or AsA). Then, elongation ratio (*n* = 6) (**B**), capillary assay (*n* = 9) (**C**), and level of contraction protein (caldesmon, tropomyosin), vimentin, and α-SMA were analyzed by Western blot (**D**), with GAPDH used as the loading control. The results are provided as means ± SD (*n* = 3), ** *p* < 0.001, *** *p* < 0.005. The blots are representative of three independent experiments. The values from control cells are marked by dashed lines.

Next, CAF-like cells maintained in medium obtained from cancer cells (Figure 5A) were treated with vincristine with or without NSAIDs—IBU or AsA (Figure 5), which are well described as immunomodulators [16]. The CAFs-CM1 + VIN + IBU or AsA or CAFs-CM2 + VIN + IBU or AsA medium samples demonstrated inhibition of vincristine-stimulated secretion of IL-6 and TGF-βs. The differences ranged from 0.5 times in IL-6 to about 2.5 times in TGF-β1 (Figure 5A). The following analysis revealed that alteration of the profile of secreted cytokines inhibited vincristine-dependent EndMT. Additionally, the control of loading the blots was labeled by Ponceau Red. The morphological analysis demonstrated that NSAIDs blocked vincristine-dependent cell elongation (Figure 5B, Figure S2A). The HMEC-1 cells grown in co-CM1 + VIN, co-CM2 + VIN, CAFs-CM1 + VIN, or CAFs-CM2 + VIN enriched in one of the NSAIDs (AsA or IBU) displayed similar capillary formation to the control cells, suggesting that AsA or IBU are able to prevent vincristine-induced CAF-like formation. Furthermore, cells treated with CAFs-CM1 + VIN + IBU or AsA or CAFs-CM2 + VIN + IBU or AsA formed capillaries about 10% longer, suggesting greater angiogenesis ability (Figure 5C, Figure S2B). Western blot analysis of contraction proteins (caldesmon and tropomyosin) and other mesenchymal marker levels such as vimentin and α-SMA showed that both AsA and IBU inhibit the increase in the protein levels observed following vincristine induction (Figure 5D). We did not observe the increase of contraction proteins and vimentin, and α-SMA level after only AsA or IBU treatment. Additionally, NSAIDs treatment (AsA and IBU) did not induce the EndMT process (Figure S3).

### 2.4. NSAIDs Regulate EndMT by Modulation of Microtubule Polymerization and Decrease the Level of TUBB3 in the Microtubules

**Figure 6:**
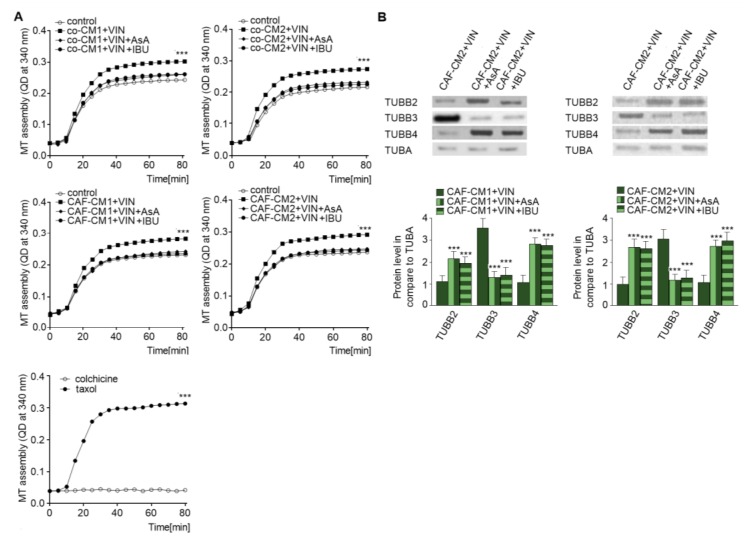
NSAIDs decrease microtubules polymerization through modulation of the composition of beta subunits in microtubules. The polymerization ability was analyzed in microtubules isolated from coculture of CAF-like cells and colon cancer, or CAF-like cells maintained in CM colon cancer cells and treated with vincristine and NSAIDs (IBU or AsA) (**A**). Additionally, controls of microtubules polymerization with taxol and depolymerization with colchicine were conducted and are shown in the bottom graph (representative). Next, the levels of TUBB2, TUBB3, and TUBB4 were determined by Western blot assay in microtubules fraction of CAF-like cells (**B**). The protein levels were normalized to TUBA. The results are provided as means ± SD (*n* = 3); *** *p* < 0.005. The blots are representative of three independent experiments.

As vincristine acts as a regulator of microtubule polymerization [17] and NSAIDs are able to protect against the effects of vincristine, the next stage of the study examined the role of AsA or IBU in microtubule proliferation. Cytoskeleton tubules isolated from CAF-like cells treated with vincristine and NSAIDs (AsA or IBU) were found to display slower polymerization (Figure 6A). Polymerization with taxol and colchicine was analyzed as the control (Figure 6A, panel right). To better determine the levels of particular tubulin subunits in microtubules (Figure 6B), Western blot assay was performed. The results revealed an increase of TUBB4B and TUBB2 levels and lower TUBB3 levels in the cells grown in the medium enriched in NSAIDs (CAFs-CM1-VIN + IBU or AsA or CAFs-CM2 + VIN + IBU or ASA) compared to cells treated with vincristine (CAFs-CM1-VIN or CAFs-CM2 + VIN) (Figure 6B).

### 2.5. Alterations of TUBB2, TUBB3, and TUBB4 affect IL-6 and TGF-βs Secretion Through Modulation of Microtubules Polymerization Dynamics

**Figure 7:**
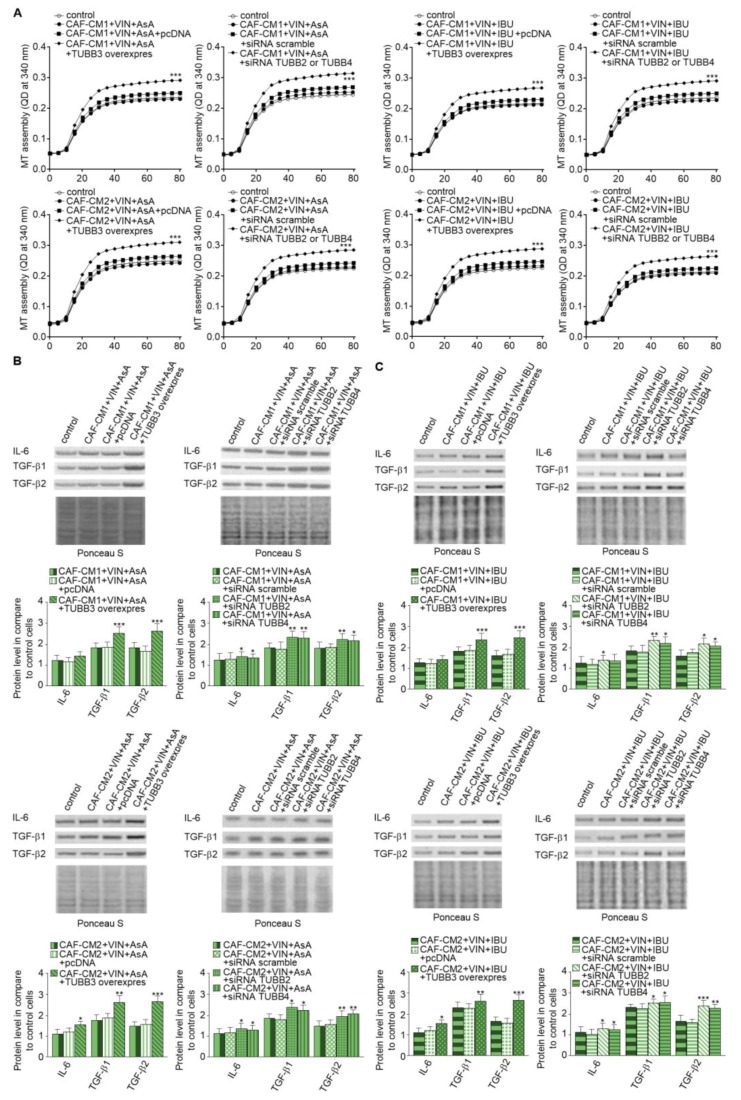
Modulation of β-tubulins level alters IL-6 and TGF-βs secretion through regulation of microtubules polymerization. The polymerization ability was analyzed in microtubules isolated from CAF-like cells maintained in CM colon cancer cells treated with vincristine and one of the NSAIDs—IBU or AsA where TUBB was overexpressed (TUBB3 overexpres) and TUBB2 or TUBB4 were silenced (siRNA TUBB2 or siRNA TUBB4) (**A**). Next, the level of TGF-βs (TGF-β1 and TGF-β2) and IL-6 was analyzed in medium from cells treated with NSAIDs (AsA or IBU) by Western blot. The results are provided as means ± SD (*n* = 3); * *p* < 0.05, ** *p* < 0.001, *** *p* < 0.005. The blots are representative of three independent experiments (**B**,**C**). As the control, an empty vector (pcDNA) or nonspecific fragments (siRNA scramble) were used. As the control of loading, the Ponceau Staining was shown.

Finally, we focused on research on the role of particular β-tubulin expressions in the regulation of IL-6 and TGF-βs released. The analysis of microtubules polymerization isolated from the CAF-like cells treated with VIN and IBU or ASA where TUBB3 was overexpressed showed that it increases the polymerization ability in comparison to microtubules obtained from cells transfected with empty vector as well as controls nontransfected cells. In contrast, TUBB2 or TUBB4 evokes the opposite effect. It has been determined that silencing of particular β-subunits in the CAF-like cells maintained under VIN and NSAIDs pressure induces faster polymerization microtubules in vitro compared to cells where nonspecific sequences were used for silencing assay (scramble) (Figure 7A). Finally, correlations have been observed between modulation of particular tubulin class beta and ability to secrete IL-6 and TGF-βs in CAF-like cells treated with VIN and one of the analyzed NSAIDs. In particular, overexpression of TUBB3 and silencing of TUBB2 and TUBB4 affect the increase of IL-6 and TGF-βs release by CAF-like cells (Figure 7B). Ponceau Red was used for loading control analysis. 

## 3. Discussion

Vincristine is widely used in the clinical treatment of leukemia, lung cancer, and other malignant tumors [18,19,20,21]. It is the most commonly administered chemotherapy agent in the clinical treatment of colon cancer, especially in the metastatic stages [22]. Nevertheless, numerous studies indicate that the tumor gradually acquires resistance to vincristine during therapy [23,24]. Previous analyses have demonstrated that the molecular mechanism in tumor cells underlying vincristine resistance is complex and involves a number of genes, including insulin-like growth factor binding protein 7 and multidrug resistance protein 1, in addition to long noncoding RNA [25,26,27]. Although chemotherapeutics, including vincristine, might act not only on the tumor but also on the stromal cells, that effect has been usually ignored. According to that, it has been postulated that resistance to vincristine might be also mediated by immunomodulation of stromal cells, mainly CAFs [28].

CAFs are described as the main regulators of cancer progression and metastasis [29]. They are able to transform cancer from the preinvasive stage to a more invasive form by increasing the migration and invasion ability during the EMT. Those processes are mediated by two CAF-released factors: TGF-βs and IL-6 [29]. Like other stromal cells, CAFs interact with cancer cells, regulating their proliferation and invasive capabilities [30]. On the other hand, cancer cells modulate the activities of CAFs, which may be manifested by a change in the profile of secreted factors. These alterations affect cancer development and progression, including metastatic transformation, and play an intermediary role in chemoresistance [31].

The present study focuses on the role played by the interaction between colon cancer stromal cells in the modulation of vincristine-dependent colon cancer resistance. Therefore, different cellular models were established to study the role of vincristine in the modulation of interaction between colon cancer cells and CAF-like cells. We observed that exposure of endothelial cells to conditioned medium obtained from vincristine treatment cocultured of cancer cells and CAF-like cells can induce a CAF phenotype. A higher incidence of CAF-like cell formation through EndMT process was characterized by cell shape elongation, a decrease of endothelial markers, proliferation rate, and ability to form tubes which characterize endothelial function [32]. On the other hand, an increase in mesenchymal and CAFs markers was observed. It is known that intercellular crosstalk between tumor and the stromal cells, of which CAFs are the most abundant, promotes cancer progression, metastasis, and resistance to anticancer therapy [32]. It is also well established that the interaction between tumor and the microenvironment that surrounds the tumor cells depends on cancer malignancy [32]. Therefore, the study examined whether colon cancer stage progression affects vincristine-dependent CAF formation. Interestingly, no such relationship was observed, which suggests that the phenomenon of CAF formation might occur following vincristine treatment regardless of the stage of tumor progression. Our more accurate studies demonstrated that vincristine-dependent CAF formation is the effect of paracrine stimulation by colon cancer cells. This process did not occur when vincristine-treated cancer cells were stimulated with the medium from the tumor cells only. Therefore, under unfavorable conditions, vincristine-based therapy might, in fact, induce cancer invasion and metastasis by increasing the number of CAFs in the cancer microenvironment rather than acting as a cure.

Studies investigating CAF function found them to be a source of secreted factors other than those involved in paracrine signaling or activation of CAFs in autocrine loops, thereby forming the CAF phenotype [33]. Our present findings show that vincristine treatment of cocultured cancer and CAF-like cells resulted in increased secretion of IL-6 and TGF-βs, both known as EMT inducers [34]. Additionally, TGF-β2 has been found to be the main inductor of EndMT in the microvessel endothelium present in the cancer niche [35,36,37]. EndMT is a critical source of CAF formation which enhances cancer migration and invasion, leading to tumor metastasis.

NSAID-based therapy plays an important role in the treatment of invasive cancer. Increasing numbers of studies have shown the positive effect of these drugs on many solid tumors through the modulation of the inflammatory effect induced by stromal cells [38,39,40,41,42]. Nevertheless, the molecular mechanisms of NSAIDs in the prevention of metastasis is still poorly understood. Recently it has been suggested that IBU might modulate the microtubule polymerization in fibrotic diseases [8]. Our findings indicate that cells treated with AsA or IBU (the most commonly used NSAIDs) displayed a decreasing vimentin-induced EndMT ability. We observed an increase of capillary formation ability, inhibition of cell elongation, and lack of modulation of endothelial and mesenchymal markers expressions. What is more, we observed that NSAIDs inhibited vincristine-dependent secretion of IL-6 and TGF-βs. Assuming that the endothelium plays a critical role as the main source of CAFs, inhibition of the EndMT would prevent CAF formation and, ultimately, tumor metastasis. 

**Figure 8:**
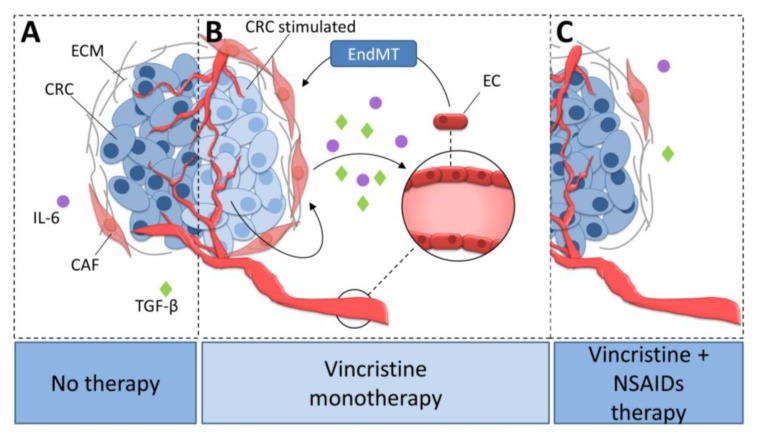
The role of NSAIDs in the inhibition of vincristine-dependent CAFs formation in colon cancer cells. (**A**) CAFs stimulate colon cancer development. (**B**) Vincristine therapy induce EndMT in microvessel ECs through the increasing release of TGF-βs and IL-6 from CAFs. (**C**) The NSAIDs inhibit the vincristine-dependent secretion of TGF-βs and IL-6 from CAFs. Colon cancer cells stimulated with vincristine promote CAFs secretion ability. Activated CAFs increasingly release IL-6 and TGF-βs and therefore stimulate CAFs formation from EC cells through EndMT process. NSAIDs prevent CAFs formation through inhibition of IL-6 and TGF-βs secretion. CRC, colon cancer cell; CRC stimulated, colon cancer cell stimulated with vincristine; CAF, cancer-associated fibroblast; EC, endothelial cell; ECM, extracellular matrix; TGF-β, transforming growth factor-β; IL-6, interleukin-6; EndMT, endothelial-to-mesenchymal transition.

Vincristine is a microtubule rearrangement modulator that works by binding to the tubulin and destabilizing microtubule structure [43]. Based on this study and our observations that AsA and IBU inhibited the effect of vincristine-dependent increasing secretion of IL-6 and TGF-βs, we suppose that analyzed NSAIDs might regulate microtubule rearrangement and function (Figure 8). The addition of NSAIDs to vincristine-based treatment resulted in slower microtubule polymerization. An analysis of the level of beta-tubulin isoforms showed a decrease of TUBB3 and increase of TUBB2 and TUBB4 in the microtubules after NSAID treatment. It has previously been shown that the dimers TUBA1TUBB2 and TUBA1TUBB4 located in microtubules promote slower microtubule polymerization and the formation of more stable tubes. In contrast, higher levels of TUBA1TUBB3 in microtubule structures increase their polymerization rate. Faster microtubule polymerization and higher TUBB3 levels are observed during EMT or EndMT. In contrast, a decrease in TUBB3 level or an increase of TUBB4 and TUBB2 result in the inhibition of mesenchymal transdifferentiation.

Our study demonstrates that vincristine monotherapy could be ineffective in the treatment of colorectal cancer. What is more, it seems that monotherapy based on vincristine may favor metastasis by increasing the number of CAFs in the tumor niche. The results of the presented analysis suggest that combining vincristine therapy with anti-inflammatory treatment can prevent these undesirable effects.

## 4. Materials and Methods

### 4.1. Reagents

Unless specified otherwise, all reagents were obtained from Sigma-Aldrich (Steinheim, Germany).

### 4.2. Cell Cultures

CAF-like cells were obtained by TGF-β2 stimulation of human microvascular endothelial cells (HMEC-1) as described previously [35,36,37]. HMEC-1 were cultured in MCDB131 (Life Technologies, Paisley, UK) medium supplemented with 10% heat-inactivated fetal bovine serum (FBS) (Life Technologies) or with one of CM: co-CM1, co-CM2, co-CM1 + VIN, co-CM2 + VIN, CAFs-CM1, CAFs-CM2, CAFs-CM1 + VIN, CAFs-CM2 + VIN, or CAFs-CM. All lines were maintained at 37 °C in a humidified 5% CO_2_ atmosphere. CM was recovered from colon cancer cells grown for 72 h. In some experiments, the cells were treated with vincristine (5 nM) or NSAIDs: 2500 µM aspirin (AsA) and 400 µM ibuprofen (IBU). The cells were harvested by 0.05% trypsin-EDTA and washed with phosphate-buffered saline (PBS).

### 4.3. Conditioned Media

Coculture of CAF-like cells and colon cancer cells were obtained by mixing 50,000 colon cancer cells/cm^2^ and 4000 CAF-like cells/cm^2^ maintained in DMEM medium (Life Technologies, Paisley, UK) supplemented with 10% heat-inactivated fetal bovine serum (FBS) (Life Technologies). After two days, the conditioned medium (CM) was collected, centrifuged to remove cells, and frozen. Two types of coculture were prepared with preinvasive LS180 cells (co-CM1) or invasive LoVo (co-CM2) cells. In some experiments, cells were treated with vincristine during the final 24 h (co-CM1 + VIN or co-CM2 + VIN).

In addition, CM from two-day culture of colon cancer (50,000 cells/cm^2^) was added to CAF-like cells (4000 cells /cm^2^) in a 1:3 ratio. The cells were maintained in the medium over the next two days and then the CM was collected from CAF-like cells as described above. As in the case of coculture, two types of cancer cells were used: preinvasive LS180 cells (CAFs-CM1) and invasive LoVo (CAFs-CM2) cells. As previously, the CAF-like cells were treated with vincristine during the final 24 h (CAFs-CM1 + VIN or CAFs-CM2 + VIN). Additionally, CM from two-day culture of CAF-like cells (4000 cells/cm^2^) was added to LS180 (colon-CM1) or LoVo (colon-CM2) colon cancer cells (50,000 cells /cm^2^) in a 1:3 ratio. The cells were maintained in the medium over the next two days; following this, the CM from the colon cancer culture was centrifuged and collected for the next experiments. As previously, CAF-like cells were treated with vincristine during the final 24 h (CAFs-CM1 + VIN or CAFs-CM2 + VIN). In some experiments, the cells (coculture, CAF-like cells maintained in CM from colon cancer cells, or colon cancer cells grown up in CM from CAF-like cells) were treated with NSAIDs (ibuprofen—IBU or aspirin—AsA, respectively) for the last three days. In some cases, CM supplemented with TGF-β2 (CAF-CM) was collected from CAF-like cells obtained from HMEC-1 (a gift from Prof. Kathryn Keller, Centers for Disease Control and Prevention, Atlanta, GA, USA).

### 4.4. Cell Proliferation Assay

For the proliferation assay, the cells were seeded on a 96-well flat-bottom plate. The 24-h and 48-h cells were treated with appropriate factors. The cells were gently washed with warm PBS, fixed with Carnoy’s solution every six days, dried, and stained with DAPI (4 μg/mL). The cells were then gently washed three times with 3× distilled water and dissolved in 10 mM EDTA, pH 12. The fluorescence was directly measured using a plate reader (Victor, Perkin Elmer) at a wavelength of 485 nm after a 30-minute incubation at 37 °C. The growth curves were determined by GraphPad Prism software.

### 4.5. Cell Morphology

The shape of the maintained analyzed cells was observed under fluorescence microscopy (Olympus, San Jose, CA, USA) and five representative images were captured by a digital camera (Olympus, San Jose, CA, USA). At least 50 cells in each experimental condition were then measured to calculate the elongation ratio, that is, the ratio of the longer to the shorter axis, using ImageJ software (NIH, Bethesda, MD, USA).

### 4.6. Tube Formation Assay

To analyze in vitro capillary-like tube formation, Matrigel™ was coated onto a 24-well plate, then cells (5 × 10^4^/mL) in complete cell culture medium were seeded onto precoated plates. After an eight-hour incubation period, at 37 °C in 5% CO_2_, the cells were observed under a phase-contrast microscope and representative images captured (Olympus microscope). The formation of capillary tube-like networks and their total length were examined using ImageJ software and are shown in the graph.

### 4.7. Microtubule Isolation

Microtubule proteins were isolated as described previously [44]. The cell pellets were homogenized in PB buffer (0.1 M K-PIPES (pH 6.8), 0.5 mM MgCl_2_, 2 mM EGTA, 0.1 mM EDTA, 0.1% (*v*/*v*) β-mercaptoethanol, 1 mM ATP with PhosStop phosphatase inhibitor and cOmplete Protease Inhibitor Cocktail) before centrifuging (100,000× *g*, 60 min, 4 °C). Next, the cytosolic supernatants were collected, mixed with a half volume of 100% glycerol preheated at 37 °C with ATP and MgCl_2_ (concentrations of 3.5 mM), and polymerized for 60 min at 37 °C. The pellets were collected, centrifuged (100,000× *g*, 45 min, 37 °C), resuspended with ice-cold PB buffer, and depolymerized on ice for 30 min. This process was repeated twice. Next, the cell proteins were extracted (15 min at room temperature) with 0.5% NP-40 in a microtubule stabilization buffer containing 20 mM Tris, pH 6.9, 0.5% (*v*/*v*) NP-40, 2 mM glycerol, 10% (*v*/*v*) DMSO, 1 mM MgCl_2_, 2 mM EGTA, 200 mM sodium orthovanadate, 1 mM phenylmethylsulfonyl fluoride (PMSF) and PhosStop phosphatase inhibitor and cOmplete Protease Inhibitor Cocktail. The detergent-insoluble material was pelleted by centrifugation (15,000× *g*, 10 min, RT) and soluble extracts were used for Western blot with appropriate antibodies.

### 4.8. Western Blot Assay

Briefly, the harvested cells were lysed in M-PER (Mammalian Protein Extraction Reagent) with Halt Protease Inhibitor Cocktail (Thermo Scientific Pierce, Rockford, IL, USA) according to the manufacturer’s protocol, and the obtained extracts were collected, aliquoted, and stored at −80 °C. In some experiments, CMs were also collected as described above in point 4.3. Protein quantification was performed with a BCA Protein Assay Kit according to the manufacturer’s protocol. The lysates (30 μg) or CM were separated by electrophoresis and electroblotted as described previously and analyzed by Western blot assay [45]. Protein levels were normalized using an appropriate loading control (GAPDH or tubulin-α). In the experiments where protein from CM was analyzed, Ponceau Staining blots were used as the loading control. In particular experiments, the blots were incubated with primary antibodies recognizing α-SMA, tropomyosin, caldesmon, vimentin (cell signaling), TUBA, TUBB2, TUBB3, TUBB4, GAPDH and IL-6, TGF-β1 or TGF-β2. Then, appropriate secondary anti-rabbit or anti-mouse horseradish peroxidase-conjugated antibodies (Dharmacon) were used.

### 4.9. Microtubule Polymerization

Briefly, the isolated tubulin proteins were suspended in G-PEM buffer containing 80 mM PIPES, 2 mM MgCl_2_, 0.5 mM ethylenediaminetetraacetic acid, and 1.0 mM GTP (pH 6.9) and 5% glycerol in a 96-well plate, and their absorbance was measured at 340 nm from 0 to 80 min (Synergy H4 multimode microplate reader BioTek (Winooski, VT, USA). The tubulin polymerization assay was performed in three independent experiments [46].

### 4.10. Statistical Analysis

The results are presented as the mean of at least three independent experiments ± standard error. The statistical significance of the differences between the experimental conditions was determined by one-way ANOVA followed by Tukey’s test (GraphPad Prism Software, 8.0.0 for Windows, San Diego, CA, USA). Differences between means were considered significant when *p* < 0.05.

## Supplementary Materials

Supplementary materials can be found at https://www.mdpi.com/1422-0067/20/8/1941/s1.

## Abbreviations

CAFs	Cancer-Associated Fibroblasts
EndMT	Endothelial–Mesenchymal Transition
HMEC-1	Human Microvascular Endothelial Cells
TGF-β	Tumor Growth Factor-β
CM	Conditioned Medium
TUBA	Tubulin-α
TUBB	Tubulin-β
ECM	Extracellular Matrix
VIN	Vincristine
IBU	Ibuprofen
AsA	Aspirin
IL-6	Interleukin 6
