# Supplementary material for: Nonsteroidal Anti-Inflammatory Drugs Prevent Vincristine-Dependent Cancer-Associated Fibroblasts Formation
Source: Int J Mol Sci. 2019 Apr 20;20(8):1941. doi: 10.3390/ijms20081941 (PMC6515011; doi:10.3390/ijms20081941)
Supplement: Supplementary file 1 [file ijms-20-01941-s001.pdf]

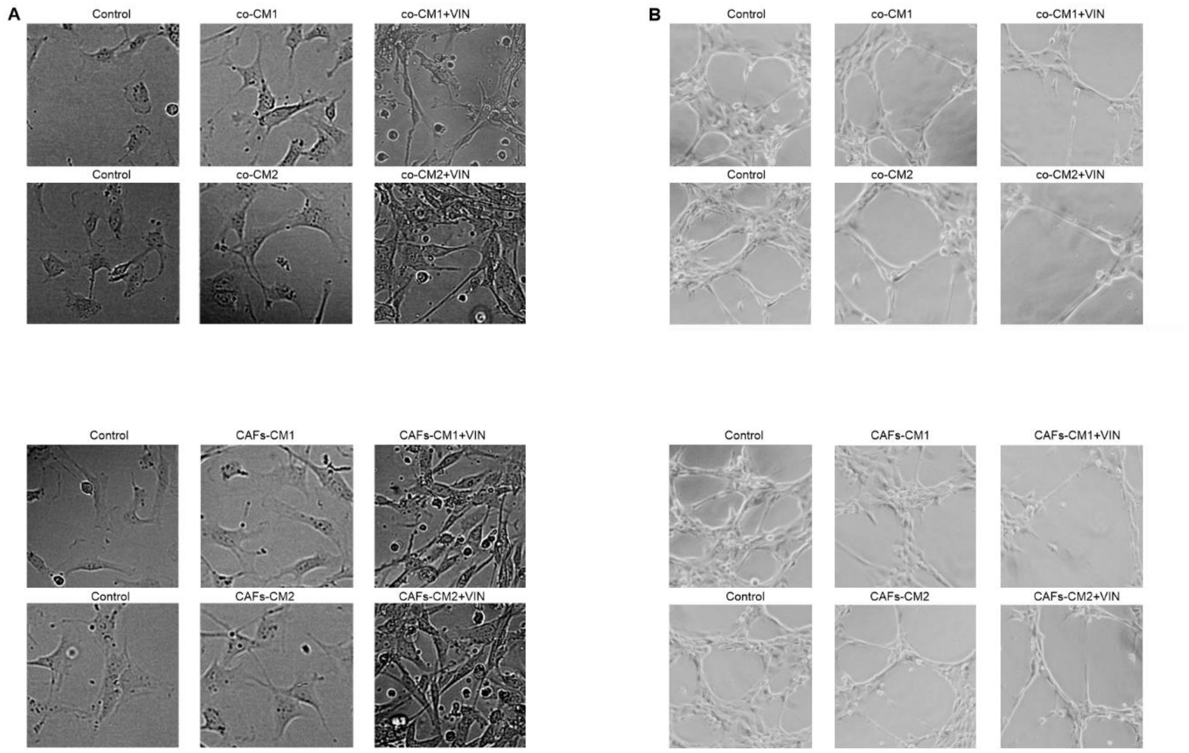

Figure S1. Mesenchymal transdifferentiation in HMEC-1 is modulated by vincristine-treated CAF-like cells. HMEC-1 cells were cultured in medium supplemented with CM isolated from co-culture of CAF-like cells and colon cancer (LS180 - co-CM1 or LoVo – co-CM2) and treated, if necessary, with vincristine (+VIN). (A) representative images of cell shape (B) representative images of capillary formation.

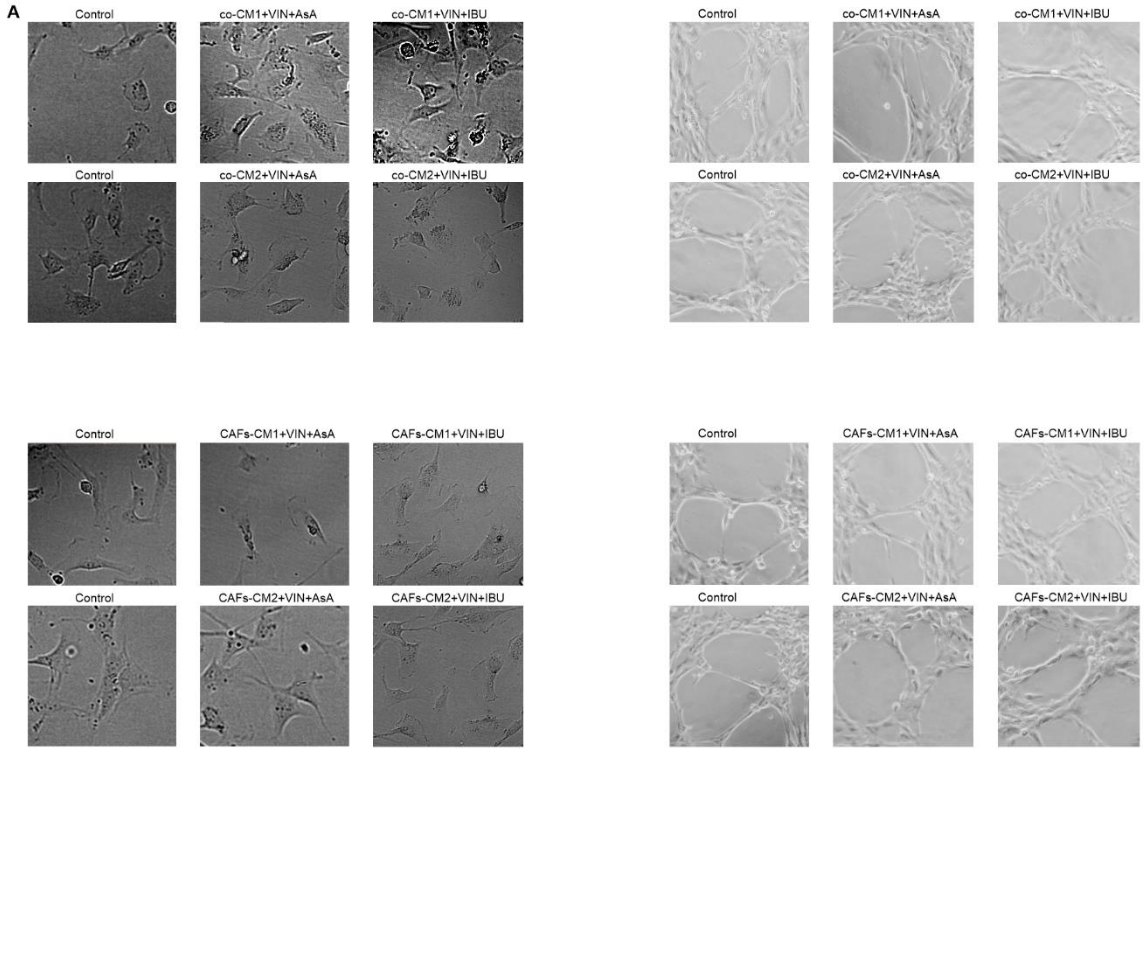

Figure S2. Mesenchymal transdifferentiation in HMEC-1 is modulated by vincristine-treated CAF-like cells. HMEC-1 cells were cultured in medium supplemented with CM isolated from CAF-like cells maintained in CM colon cancer cells (LS180 - CAFs-CM1 or LoVo – CAFs-CM2) and treated, if necessary, with vincristine (+VIN). (A) representative images of cell shape (B) representative images of capillary formation.

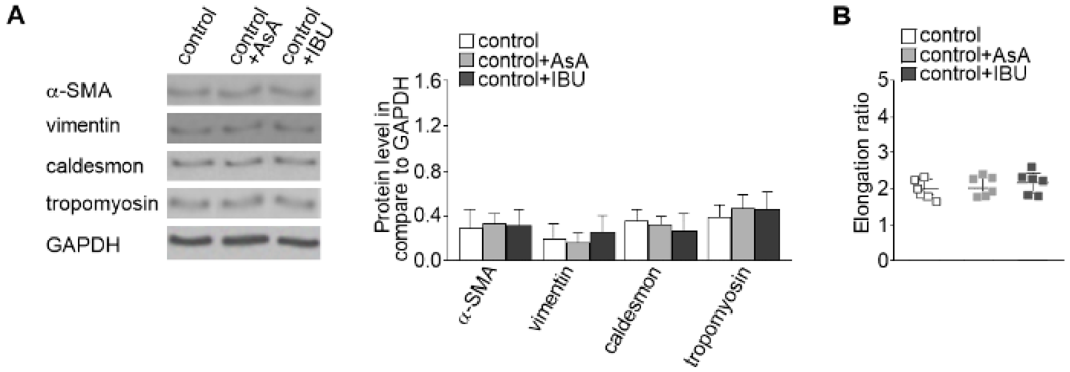

Figure S3. NSAIDs non-induce the EndMT in HMEC-1 cells. The HMEC-1 cells were cultured in medium and treated, if necessary, with NSAIDs (IBU or AsA). Then, levels of contraction proteins (caldesmon, tropomyosin), vimentin and α-SMA were analyzed by Western blot (A). The GAPDH was used as the loading control. The results are provided as means ± SD (N = 3) *** $p < 0.005$. The blots are representative of three independent experiments. The values from control cells were marked by dashed lines. Next, elongation ratio (N=6) (B) were measured.
